# Human Cytomegalovirus Genome Diversity in Longitudinally Collected Breast Milk Samples

**DOI:** 10.3389/fcimb.2021.664247

**Published:** 2021-04-16

**Authors:** Jasper Götting, Katrin Lazar, Nicolás M. Suárez, Lars Steinbrück, Tabea Rabe, Rangmar Goelz, Thomas F. Schulz, Andrew J. Davison, Klaus Hamprecht, Tina Ganzenmueller

**Affiliations:** ^1^ Institute of Virology, Hannover Medical School, Hannover, Germany; ^2^ Institute for Medical Virology and Epidemiology of Viral Diseases, University Hospital Tuebingen, Tuebingen, Germany; ^3^ MRC-University of Glasgow Centre for Virus Research, Glasgow, United Kingdom; ^4^ Department of Neonatology, University Children’s Hospital, Tuebingen, Germany

**Keywords:** cytomegalovirus, breast milk, genomes, strains, diversity, genotyping, immunocompetent, lactation

## Abstract

Reactivation and shedding of human cytomegalovirus (HCMV) in breast milk during lactation is highly frequent in HCMV-seropositive mothers. This represents a key transmission route for postnatal HCMV infection and can lead to severe disease in preterm neonates. Little is known about HCMV strain composition or longitudinal intrahost viral population dynamics in breast milk from immunocompetent women. We performed HCMV-specific target enrichment and high-throughput sequencing of 38 breast milk samples obtained in Germany between days 10 and 60 postpartum from 15 mothers with HCMV DNA lactia, and assembled HCMV consensus sequences *de novo*. The genotype distribution and number of HCMV strains present in each sample were determined by quantifying genotype-specific sequence motifs in 12 hypervariable viral genes, revealing a wide range of genotypes (82/109) for these genes in the cohort and a unique, longitudinally stable strain composition in each mother. Reactivation of up to three distinct HCMV strains was detected in 8/15 of mothers, indicating that a representative subset of the woman’s HCMV reservoir might be locally reactivated early during lactation. As described previously, nucleotide diversity of samples with multiple strains was much higher than that of samples with single strains. Breast milk as a main source of postnatal mother-to-infant transmission may serve as a repository for viral diversity and thus play an essential role in the natural epidemiology of HCMV.

## Introduction

Infections with human cytomegalovirus (HCMV; species *Human betaherpesvirus 5*), which is a large, enveloped virus from subfamily *Betaherpesvirinae* of family *Herpesviridae*, are highly prevalent in people worldwide and result in life-long viral persistence (Boppana and Fowler, 2007; Davison, 2010; Manicklal et al., 2013). HCMV infections are acquired during all stages of life *via* numerous transmission routes (vertically and by various bodily fluids), and mostly remain asymptomatic in immunocompetent individuals. However, HCMV infection can lead to severe complications in transplant recipients, congenitally infected children, and upon postnatal transmission (Balfour, 1979; Manicklal et al., 2013; Kotton et al., 2018; Krishna et al., 2019).

Postnatal HCMV infections of preterm or very low birth weight (VLBW) infants pose the risk of severe disease with around 4% fatality (Martins-Celini et al., 2016; Hamprecht and Goelz, 2017; Osterholm and Schleiss, 2020). A main source of postnatal HCMV infections is the feeding of raw breast milk (BM), which is considered to be the principal transmission route (Stagno et al., 1980; Doctor et al., 2005; Hamprecht et al., 2008). Local HCMV reactivation during lactation is observed in up to 97% of HCMV-IgG-seropositive mothers, lasting around three months postpartum, and viral DNA can be detected by PCR-based methods in milk whey (DNA lactia) (Hamprecht et al., 2001; Lazar et al., 2020). This unique reactivation mode at the onset of lactation is limited to a local reactivation in the mammary glands, whereas HCMV DNA is not detected in the mother’s blood during this period (Mosca et al., 2001; Numazaki et al., 2001; Hamprecht et al., 2003).

Given its role in the natural transmission of HCMV and the resulting health burden on preterm and VLBW infants, BM is an important material to study. However, detailed studies analyzing whole HCMV genomes from BM are few, and little is known about viral strain composition and diversity, or about the longitudinal population dynamics of HCMV in BM during lactation-induced reactivation (Hamprecht et al., 2008; Hamprecht and Goelz, 2017; Suárez et al., 2019a). The diversity of HCMV genomes (236 kbp) is an evolutionary result of the generation of hypervariable genes coupled with a general loss of genetic linkage due to recombination (Bradley et al., 2008; Sijmons et al., 2015; Lassalle et al., 2016). The advent of target-enrichment as a prelude to sequencing library preparation has enabled high-throughput sequencing (HTS) of HCMV genomes directly from clinical specimens with less bias and higher coverage compared to metagenomic and amplicon-based approaches (Depledge et al., 2011; Houldcroft et al., 2016; Houldcroft et al., 2017; Suárez et al., 2019a; Suárez et al., 2019b). However, most research on HCMV strain composition has focused on genotyping single hypervariable genes in the context of HCMV as an important transplant pathogen, which, due to the involvement of immunosuppression, reinfection, and donor/recipient serostatus, does not represent natural transmission situations (Görzer et al., 2010; Renzette et al., 2013; Hage et al., 2017).

In this study, we explore the strain diversity and longitudinal intrahost dynamics of HCMV in BM from a cohort of healthy, immunocompetent mothers. This was accomplished using HTS of HCMV genomes by target enrichment coupled with a recently developed genotyping method that discriminates short motifs in hypervariable genes and allows single-strain infections to be distinguished from multiple-strain infections (Suárez et al., 2019b).

## Material and Methods

### Cohort and BM Specimens

BM samples originated from the BlooMil study (Lazar et al., 2020; Rabe et al., 2020) and were analyzed in collaboration with the Department of Neonatology at the University Children’s Hospital, Tuebingen. Samples (~80 ml) from HCMV-seropositive mothers of mostly preterm infants were collected within four defined time periods: 10–15, 25–30, 40–45 and 55–60 days postpartum (T1–T4). No data were available on the site of sample collection (i.e. left or right breast). Additionally, women filled out questionnaires providing data on age and number of children. Women gave informed consent, and the study was approved by the local Ethics Committee of University Hospital Tuebingen (567/2017BO2).

### BM Preparation and DNA Extraction

BM was prepared and centrifuged as described previously (Lazar et al., 2020) to separate cellular, whey and fatty fractions. HCMV DNA loads of cell-free whey fraction specimens were determined by quantitative real-time PCR (CMV-R-Gene, bioMérieux, lower limit of detection = 600 copies/ml) on a LightCycler (Roche). Whey fraction samples were archived at –80°C. Based on viral load (>1000 copies/ml) and availability of longitudinal samples, 38 specimens from 15 mothers were selected for HTS. DNA was extracted from 400 µl archived BM whey fractions and eluted in 200 µl buffer using a QIAgen DNeasy Blood & Tissue kit on a QIAcube (Qiagen) according to the manufacturer’s instructions.

### Library Preparation, HCMV-Specific Target Enrichment, and HTS

Library preparation and sequencing were performed as described previously, with minor modifications (Hage et al., 2017). DNA was fragmented by sonication, sequencing libraries were prepared (KAPA library preparation kit, KAPA Biosystems), and PCR amplification prior to target enrichment was carried out with adapter-specific primers (14 cycles). Up to 750 ng amplified DNA was target-enriched using HCMV-specific RNA baits (ELID 0659711, SureSelect XT, Agilent). HCMV-enriched libraries were indexed, amplified (14–16 cycles), multiplexed and sequenced on a MiSeq (Illumina) using a v3 Reagent Kit to generate 2 × 300 nucleotide paired-end reads.

### *De Novo* Assembly and Phylogenetic Analysis

*De novo* assembly was performed using a pipeline incorporating *fastp* (adapter trimming and read quality filtering), *Kraken2* (human read removal), *SPAdes* (contig assembly), and *Qiagen CLC Genomics Workbench 10* (contig scaffolding and subsequent read mappings) (Nurk et al., 2013; Nurk et al., 2017; Chen et al., 2018; Wood et al., 2019). Duplicate read pairs stemming from the same DNA fragment were removed using *Picard v2.3.0*, and draft genomes were polished using *GapFiller* and *Pilon* with the deduplicated reads (Boetzer and Pirovano, 2012; Walker et al., 2014; Broad Institute, 2019). The polished draft genomes were inspected by read mapping of their corresponding read sets, annotated from HCMV strain Merlin (GenBank accession no. AY446894.2) using *Geneious Prime 2020.0.3* (Biomatters), and trimmed of the inverted repeats at the genome termini. Complete, annotated HCMV consensus genomes from mothers with single-strain HCMV reactivations were deposited in GenBank (accession numbers MW528458 – MW528464), and their respective human-filtered read data sets were deposited in the European Nucleotide Archive (http://www.ebi.ac.uk/ena/data/view/PRJEB42695, sample accession numbers ERS5621307 – ERS5621320).

### Genotype Analysis and Strain Enumeration

The quality of the libraries (fraction of human reads and number of unique HCMV reads) was assessed before genotype analysis (**Table S1**). Genotyping of 12 hypervariable HCMV genes (RL5A, RL6, RL12, RL13, UL1, UL9, UL11, UL73, UL74, UL120, UL146 and UL139) was achieved using the VATK pipeline to determine the number of reads containing conserved, genotype-specific sequence motifs of 20–31 bp in the trimmed, unfiltered read datasets, as described previously (Centre for Virus Research, 2019; Suárez et al., 2019b). The number of HCMV strains present in a sample was set as the maximum number of genotypes detected for ≥2 genes, requiring ≥25 reads containing the relevant motifs and representing ≥5% of the total number of reads identified for that gene. Genotype profiles were visualized in R using *dplyr* and *ggplot2 3.3.0* (Wickham, 2016; R Core Team, 2019). The output was used to distinguish single-strain from multiple-strain reactivations.

### Variant Analysis and Calculation of Intrahost Nucleotide Diversity

Single nucleotide polymorphisms (SNPs) were called after mapping reads to the relevant consensus genome or, in cases of longitudinal samples, after mapping the reads from each time-point to the sequence derived from the first time-point. SNPs fulfilling the following criteria were considered: read depth ≥25 (deduplicated data), average base quality ≥20, forward/reverse read balance 0.3–0.5, and variant frequency (the relative frequency of a SNP at this position) ≥2%. The SNP profiles were visualized in R as described above. This depiction facilitates the identification of the approximate abundance of minor viral populations (i.e. variant frequency <50%). The genome-wide intrahost nucleotide diversity value (π) was calculated as described previously (Nelson and Hughes, 2015). Briefly, diversity *D*i at each variable position *i* in the genome was calculated based on the frequency of the four nucleotides and the coverage of deduplicated reads mapping against the *de novo* assembly of each dataset (intra-sample diversity). The sum of the diversity *D*i of all variable positions in a genome was then divided by the length of that genome to obtain π.

## Results

### HTS of HCMV From BM

Thirty-eight HCMV DNA positive BM samples from 15 mothers (median age 33 [26–49] years; n=8 already had given birth to at least one other child) were analyzed (**Table 1**). Samples from at least two time-points were sequenced for each mother, and the full set of four samples was available for four women. As a result, it was possible to evaluate the longitudinal population dynamics for all participants (**Table 1**).

**Table 1:** Clinical information on the 15 mothers included in the study, sequenced time-points and number of detected HCMV strains.

Mother	Time-points with sequence data available	Multiple strains?	Max. number of HCMV strains detected	Age (years)	Gestational age at birth (weeks + days)	First child?
BM1	T1, —, —, T4	No	1	37	25+4	Yes
BM2	T1, —, —, T4	No	1	33	39+2	No
BM3	T1, T2, T3, T4	Yes	2	30	23+6	No
BM4	T1, T2, —, —	Yes	2	33	24+2	Yes
BM5	—, T2, T3, —	No	1	30	30+2	No
BM8	—, T2, T3, —	No	1	33	29+1	Yes
BM11	—, T2, —, T4	No	1	26	28+4	Yes
BM12	T1, T2, T3, T4	Yes	2	32	27+2	No
BM13	T1, T2, T3, T4	Yes	2	49	27+6	Yes
BM14	—, T2, T3, —	Yes	2	37	34+2	No
BM15	T1, —, —, T4	Yes	2	31	31+4	No
BM16	T1, T2, T3, T4	Yes	3	37	30+6	Yes
BM18	—, T2, —, T4	No	1	38	32+4	Yes
BM33	T1, T2, —, —	Yes	3	33	27+3	No
BM34	T1, T2, —, —	No	1	36	25+3	Yes

The sequencing libraries prepared from milk whey samples had a median viral input load of 4.4 × 10^3^ copies (9.8 × 10^2^ to 2.6 × 10^5^) per library (**Table S1**). HCMV-specific target enrichment and sequencing with an average MiSeq flow cell utilization of 12% yielded an excellent human-to-viral reads ratio and sufficient reads for the assembly of consensus HCMV genomes in all 38 cases. Even after removal of duplicate reads, the median average sequencing coverage depth of the assemblies was quite high, with 339 (15–3420) deduplicated reads per position. This coverage depth correlated very well (R^2^ = 0.71) with the viral input load during library preparation (**Figure S1**). Thus, human milk whey is an excellent specimen type for HCMV genome sequencing.

### Genotype Diversity and Strain Enumeration

To determine HCMV strain diversity during natural reactivation in immunocompetent mothers, 12 hypervariable viral genes were genotyped in each sample dataset using a genotype-specific sequence motif search. A very wide genotype variety (82 out of the 109 possible genotypes) was observed within the cohort, and the intrahost genotype composition (viral haplotype) within each mother was unique. Thus, no two mothers had the same HCMV strain(s). Multiple-strain reactivations involving up to three different HCMV strains were detected in more than half of the mothers (8/15, 53%; **Table 2**), and each of the other seven women had reactivations involving a single strain only.

**Table 2:** Genotypes of hypervariable genes in BM specimens determined *via* genotype-specific sequence motifs.

Sample	Strains	RL5A	RL6	RL12	RL13	UL1	UL9	UL11	UL73	UL74	UL120	UL146	UL139
BM1_T1	**1**	1	2	6	6	6	6	6	4A	3	2A	7	4
BM1_T4	**1**	1	2	6	6	6	6	6	4A	3	2A	7	4
BM2_T1	**1**	2	4	1A	1	1	1	4	1	1A	1A	11	2
BM2_T4	**1**	2	4	1A	1	1	1	4	1	1A	1A	11	2
BM3_T1	**2**	1	2	8	4A, 8	1, 8	5	5	3B	2A	4B	13	2
BM3_T2	**1**	1	2	8	8	8	5	5	3B	2A	4B	13	2
BM3_T3	**1**	1	2	8	1, 8	8	5	5	3B	2A	4B	13	2
BM3_T4	**1**	1	2	8	8	8	5	5	3B	2A	4B	13	2
BM4_T1	**2**	3	5	1A, 8	1, 8	1, 8	2, 4	1	1	1A, 5	1A, 2A	13	2, 4
BM4_T2	**2**	3	5	1A, 8	1, 8	1, 8	2, 4	1	1, 4D	1A, 5	1A, 2A	8, 13	2, 4
BM5_T2	**1**	2	4	1A	1	1	4	1	3A	1B	3B	9	7
BM5_T3	**1**	2	4	1A	1	1	4	1	3A	1B	3B	9	7
BM8_T2	**1**	3	5	3	3	3	2	1	4A	3	1A	14	5
BM8_T3	**1**	3	5	3	3	3	2	1	4A	3	1A	14	5
BM11_T2	**1**	1	2	6	6	6	6	1	3A	1B	3B	1	3
BM11_T4	**1**	1	2	6	6	6	6	1	3A	1B	2B, 3B	1	3
BM12_T1	**2**	2	4	1B	1	1	4	1	2, 3A	2A, 2B	1A	14	5
BM12_T2	**2**	2	4	1B, 4A	1	1	4	1	2	2B, 5	1A	14	5
BM12_T3	**2**	2	4	1B	1	1	4	1	2, 3A	2B, 5	1A	14	5
BM12_T4	**1**	2	4	1B	1	1	4	1	2	2B	1A	14	5
BM13_T1	**2**	1	1	4B, 6	1, 4A, 6	6	4, 6	7	3A	1B	1B	9, 13	5
BM13_T2	**1**	1	1	6	6	6	6	7	3A	1B	1B	9	5
BM13_T3	**2**	1	1	6	1, 6	4, 6	6	7	3A	1B, 2A	1B	9, 13	5
BM13_T4	**2**	1	1, 4	6	6	1, 6	1, 6	7	3A, 3B	1B	1B, 2A	9, 13	5
BM14_T2	**1**	3	5	3	3	3	2	1	4A	3	1A	8	1A
BM14_T3	**2**	2, 3	5	1A, 3, 8	3	3	2	1	4A, 4B	3, 4	1A	8	1A, 5
BM15_T1	**2**	1	1	4B	4B	4	3, 9	1, 6	2	2B	4B	10	2, 5
BM15_T4	**2**	1, 2	1, 4	1A, 4B	1, 4B	1, 4	4, 9	1, 6	2, 3B	2A, 2B	4A, 4B	10, 13	2, 5
BM16_T1	**3**	1, 3, 1-D2-1	1, 3, 5	4B, 6	4B, 6	4, 6	6	1, 6	3B, 4A, 4C	1A, 1C, 2A, 3	1A, 1B, 4A	12, 13	1A, 2, 5
BM16_T2	**3**	1, 3, 1-D2-1	3, 5	4B, 6	4B, 6	4, 6	6	1, 6	3B, 4A, 4C	1C, 2A, 3	1B, 4A	12, 13	1A, 2, 5
BM16_T3	**3**	1, 3, 1-D2-1	3, 5	4B, 6	4B, 6	4, 6	6	1, 6	3B, 4A, 4C	1C, 2A, 3	1B, 4A	12, 13	1A, 2, 5
BM16_T4	**3**	1, 3, 1-D2-1	3, 5	4B, 6	4B, 6	4, 6	6	1, 6	3B, 4A, 4C	1C, 3	1A, 1B, 4A	2, 12, 13	1A, 2, 5
BM18_T2	**1**	1	2	6	6	6	6	1	3A	1B	1A	12	4
BM18_T4	**1**	1	2	6	6	6	6	1	3A	1B	1A	12	4
BM33_T1	**3**	1, 2	1, 2, 4	1A, 4A	1, 4A	1, 4	4, 9	1, 6	2, 3A	1B, 2A, 2B	1A, 1B, 2B	13	2, 5
BM33_T2	**3**	1, 2, 5	1, 2, 4	1A, 4A, 7	1, 4A, 7	1, 4, 7	1, 4, 9	1, 6	2, 3A, 3B	1B, 2A, 2B	1A, 1B, 2B	1, 13	2, 5
BM34_T1	**1**	6	3	1A	1	1	1	1	4A	3	2A	12	7
BM34_T2	**1**	6	3	1A	1	1	1	1	4A	3	2A	12	7

**Figure 1:**
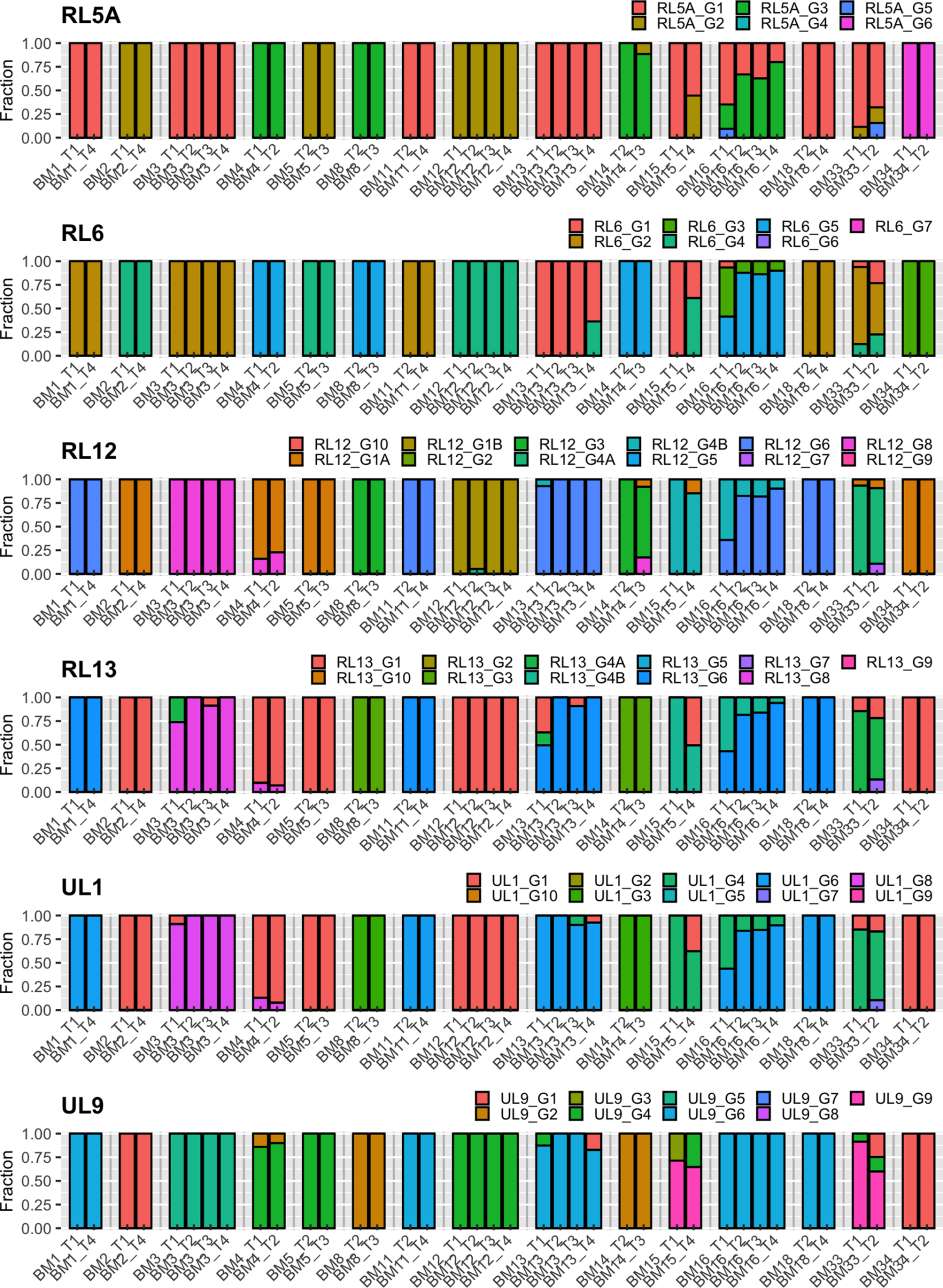
Genotype profile and relative abundance of HCMV genes RL5A – UL9 genotypes in the 38 BM specimens. Each color represents a different genotype of a hypervariable gene. Each bar represents a different sample, grouped by subject. The Y-axis indicates the fraction of genotypes in the total sequence reads for that gene in the sample.

**Figure 2:**
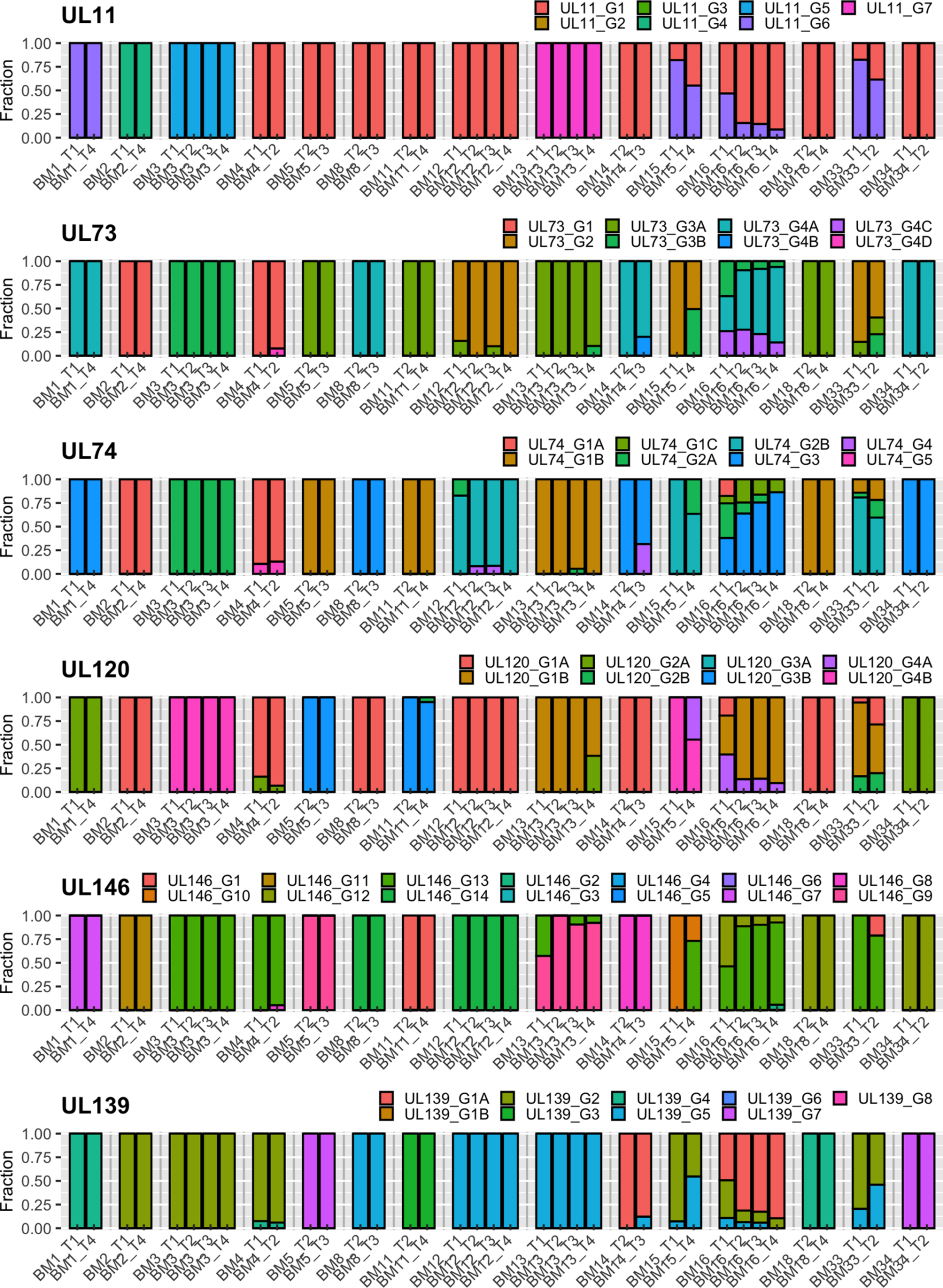
Genotype profile and relative abundance of HCMV genes UL11 – UL139 genotypes in the 38 BM specimens. Each color represents a different genotype of a hypervariable gene. Each bar represents a different sample, grouped by subject. The Y-axis indicates the fraction of genotypes in the total sequence reads for that gene in the sample.

Analysis of the intrahost genotype profile of the 12 analyzed viral genes among samples taken at longitudinal time-points for each mother revealed largely stable genotype compositions over time. Shifts in the relative proportions of individual genotypes occurred over time, but there were no drastic qualitative changes among the two or more samples obtained from each individual. This indicates that no additional genotypes possibly indicating superinfections or sequentially reactivated strains emerged at later time-points, but rather that a representative part of the latency reservoir of each host was reactivated early during lactation. In a few cases (e.g. BM15), additional genotypes seemed to have appeared at later time-points, but closer analysis revealed that these were already present at earlier time-points, but at levels below the defined reporting thresholds (**Table S1**). These genotyping results are summarized in **Table 2**, and the genotype profiles of all samples are shown in **Figures 1** and **2**. The genotype composition of a single sample containing two HCMV strains is illustrated in further detail in **Figure S3**.

### Intrahost Diversity

Intrahost nucleotide diversity (π) was calculated separately for datasets representing single strains or multiple strains. Samples containing a single strain (n=20) had π values of 6.4 × 10^–5^ (± 2.1 × 10^–4^ SD), whereas the samples representing two (n=12) or three strains (n=6) exhibited higher π values of 1.2 × 10^–3^ (± 2.4 × 10^–3^ SD) or 5.2 × 10^–3^ (± 2.5 × 10^–3^ SD), respectively (**Figure S2**). These results can be ascribed to an average of 2074 ± 1470 (range: 271–5045) or 5976 ± 332 (range: 5596–6525) variants contributing to the diversity in datasets representing two or three strains, respectively. Single-strain datasets displayed only 361 ± 420 (range: 7–1336) variants (**Figure S4**).

### Correlation of Age and Number of Children With Multiple-Strain Reactivation

No trends were observed between the presence of multiple-strain reactivation and whether a woman was below or above the median age of 33 (Fisher’s exact test, *p* = 1), or whether the child was the woman’s first (Fisher’s exact test, *p* = 0.62) (**Figure S5**).

## Discussion

BM is an important focus because of its role in postnatal HCMV transmission and the health burden imposed by postnatal HCMV infection on preterm and VLBW infants. However, studies on HCMV population dynamics at the whole genome level in BM are rare (Hamprecht et al., 2008; Suárez et al., 2019a), especially with regard to the longitudinal intrahost diversity. Analysing BM from healthy mothers with HCMV reactivation opens an opportunity to study the natural epidemiology of HCMV strains and the diversity of the genome over time in immunocompetent adults, in contrast to investigations in transplanted or HIV-infected patients who display an entirely different risk pattern for multiple HCMV infections and reinfections. This is especially pertinent, given that immunocompetent persons generally do not reactivate HCMV with DNAemia levels sufficient to allow HTS of viral genomes.

In our hands, HCMV-specific target enrichment and HTS of HCMV genomes in BM specimens resulted in excellent human-to-viral read ratios and enabled the *de novo* assembly of HCMV consensus genomes despite relatively low viral input loads. This contrasts with our experience sequencing HCMV from human plasma samples (Dhingra et al., unpublished data), where the median unique coverage depth was five times lower. This difference was possibly due to the proportion of intact genomes from virions in mammary glands, which constitute the primary locus of HCMV reactivation (Maschmann et al., 2015).

To our knowledge, this is the first description of HCMV diversity in the BM of a cohort of predominantly European, immunocompetent women. Sequencing of viral populations from longitudinally collected specimens revealed a high level of genotypic complexity with stable intrahost population compositions. Multiple-strain reactivations with up to three strains were present in 8/15 mothers (53%). No correlation was detected between the presence of multiple-strain reactivation, a woman’s age or whether the child was the woman’s first. However, as the cohort was relatively small, epidemiological conclusions should be drawn cautiously.

A previous study on HCMV strain diversity in BM specimens from HIV-positive mothers in Zambia found a similar number of different genotypes (89/109 genotypes) and reactivation of up to five strains in single individuals over a period of 4–16 weeks postpartum (Suárez et al., 2019a). The fraction of multiple-strain reactivations in our cohort was slightly lower, with 8/15 compared to 11/13 mothers in this category, and was more comparable to the 9/16 fraction observed in a cohort of transplant patients at Hanover Medical School, Germany (Hage et al., 2017). It is possible that HIV-related immunodeficiency in the Zambian cohort, as well as the generally higher seroprevalence of HCMV in developing countries, contributed to this difference (Ludwig and Hengel, 2009; Manicklal et al., 2013; Voigt et al., 2015).

HCMV strains in all 15 mothers from our cohort displayed a stable genotype composition with shifting abundance of subpopulations in multiple-strain samples but no novel emerging haplotypes that would indicate superinfection or reactivation of previously undetected strains. This is consistent with observations of two of the mothers in Zambia for whom longitudinal data were available, who also exhibited intrahost stability of HCMV populations in the initial months postpartum. Our estimates of the much lower level of intrahost diversity in single-strain reactivations in comparison with multiple-strain reactivations also corroborates the findings of previous studies in the blood compartment of immunocompromised patients (Cudini et al., 2019). This underlines the importance of controlling for the presence of multiple strains in studies of HCMV diversity in clinical specimens (Houldcroft et al., 2020; Suárez et al., 2020). Low levels of intrahost diversity in single-strain infections and reactivations stem from a small number of variants usually present at low frequency (**Figure S4**), a situation that makes diversity estimates very susceptible to outliers (**Figure S2**) (Suárez et al., 2019b).

Overall, the abundance of multiple strains reactivating in the mammary glands during lactation confirms the likelihood that BM serves not only as a major source of HCMV transmission but also as a key repository for viral diversity. We could not evaluate the transmission of HCMV to preterm infants, as sampling from neonates was not part of the study and infants under 32 weeks of gestational age were fed exclusively with short-term pasteurized BM, which would prevent transmission (Goelz et al., 2009; Klotz et al., 2018; Bapistella et al., 2019; Maschmann et al., 2019).

As HCMV reactivates strictly locally in the mammary glands during lactation and none of the women showed signs of systemic HCMV reactivation with DNAemia, strain composition and diversity among different compartments (blood and mammary glands) could not be analyzed (Lazar et al., 2020). Local reactivation during lactation is thought to be triggered by invasion of CD14+ monocytes (one site of HCMV latency) into the mammary glands (Maschmann et al., 2015). Thus, the viral populations and mixed strain reactivations that were detected likely have been present in the women prior to lactation. In the context of this reactivation mechanism the stable strain composition over time may indicate that a representative subset of the woman’s HCMV reservoir reactivates early and simultaneously during lactation.

In summary, our study provides insights into interhost and longitudinal intrahost variation of HCMV populations in BM after birth. It also demonstrates the feasibility of studying the natural diversity and transmission of HCMV in immunocompetent adults. Future work is necessary to elucidate HCMV strain dynamics during and after transmission to the infant.

## Data Availability Statement

The datasets presented in this study can be found in online repositories. The names of the repository/repositories and accession number(s) can be found below: https://www.ncbi.nlm.nih.gov/genbank/, MW528458 – MW528464. https://www.ebi.ac.uk/ena, PRJEB42695, ERS5621307 – ERS5621320.

## Ethics Statement

The studies involving human participants were reviewed and approved by Ethics Committee of University Hospital Tuebingen, Tuebingen, Germany (No. 567/2017BO2). The patients/participants provided their written informed consent to participate in this study.

## Author Contributions

JG conducted the sequencing, analyzed and interpreted the data and drafted the mansucript. LS conducted the sequencing and genome assembly. KL and TR collected samples and data, and extracted breast milk DNA. NS and AD developed the genotyping tools, conducted genome assembly and reviewed the manuscript. TS and RG contributed to conception of the study, and reviewed the manuscript. KH contributed to the study design and sample selection and reviewed the manuscript. TG designed and supervised the study, analyzed and interpreted data and reviewed the manuscript. All authors contributed to the article and approved the submitted version.

## Funding

This study was funded by the Deutsche Forschungsgemeinschaft (DFG, German Research Foundation) in Collaborative Research Centre 900 (project Z1) and under Germany’s Excellence Strategy – EXC 2155 “RESIST” – Project ID 39087428. JG was supported by the graduate program “Infection Biology” of the Hanover Biomedical Research School (HBRS). KL was supported by “Sonderlinie Medizin: Verbundprojekt Freiburg-Tuebingen-Ulm” by the Ministry of Science and Art of Baden Wuerttemberg, grant number [KL 25280-0]. TR was supported by a medical thesis grant from IZKF Promotionskolleg, University Hospital Tuebingen [201802-23]. AD and NS were supported by Medical Research Council program grant MC_UU_12014/3. We acknowledge support by the Open Access Publishing Fund of the University of Tuebingen.

## Conflict of Interest

The authors declare that the research was conducted in the absence of any commercial or financial relationships that could be construed as a potential conflict of interest.
