# Supplementary material for: Human Cytomegalovirus Genome Diversity in Longitudinally Collected Breast Milk Samples
Source: Front Cell Infect Microbiol. 2021 Apr 16;11:664247. doi: 10.3389/fcimb.2021.664247 (PMC8085339; doi:10.3389/fcimb.2021.664247)
Supplement: Supplementary file 1 [file DataSheet_1.docx]

Supplementary Material

# Supplementary Figures


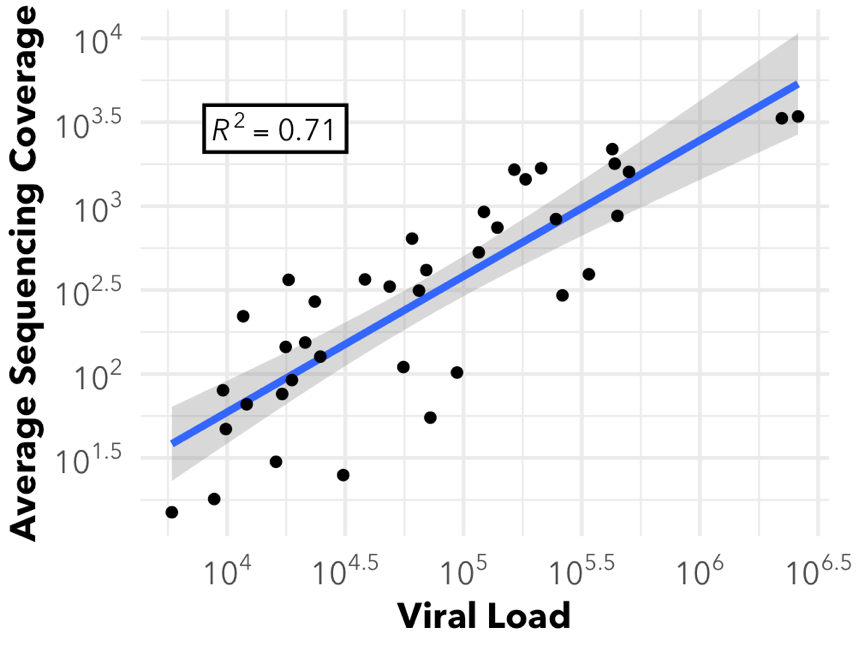


**Supplementary Figure 1.** Correlation of viral load in BM specimens with average sequencing coverage depth (deduplicated reads/nucleotide). Viral load (copies/ml) was determined by qPCR with a lower detection limit of 600 copies/ml. Coverage depth was calculated by mapping deduplicated reads to the relevant consensus sequence. Calculation of R^2^ and visualisation were performed in R.


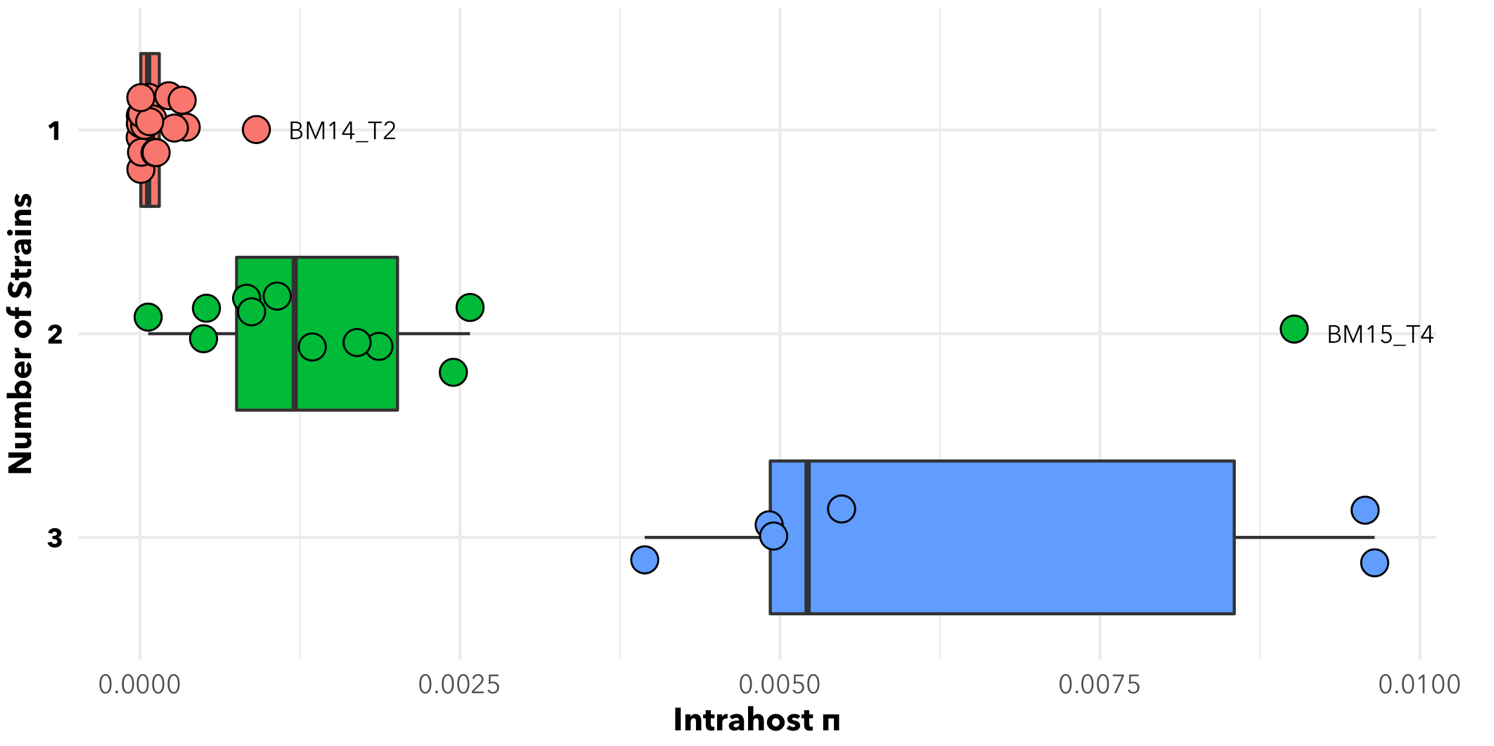


**Supplementary Figure 2.** Relationship between intrahost nucleotide diversity (π) and number of detected HCMV strains. Each dot represents a single sample, and the two outliers are named.


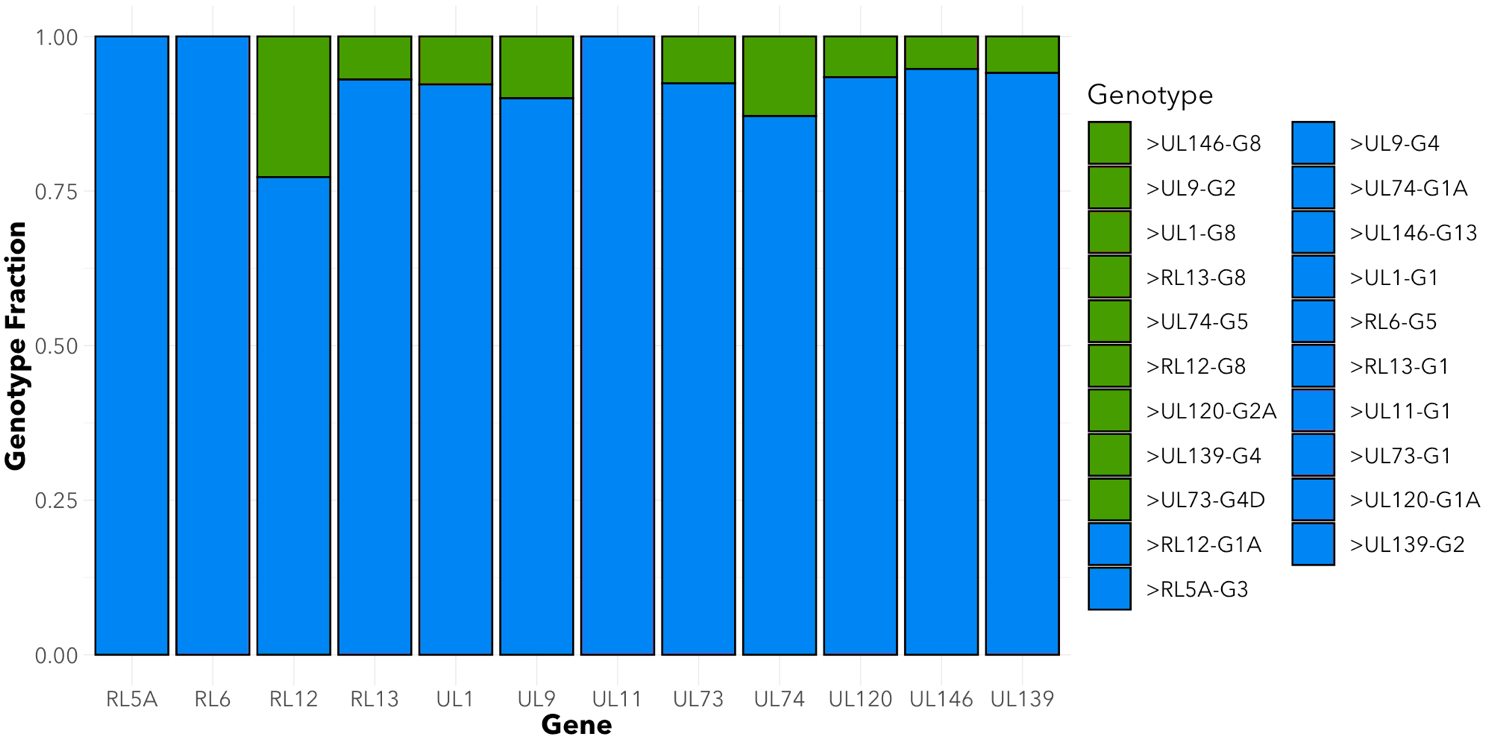


**Supplementary Figure 3.** Genotype profile of a single BM sample (BM4_T2) containing a major (blue) and a minor (green) strain. Each stacked bar represents the genotypes’ fraction of the total reads for that gene. Since these two strains have the same genotypes in RL5A, RL6, and UL11, these genotypes make up 100 % of those gene’s reads.


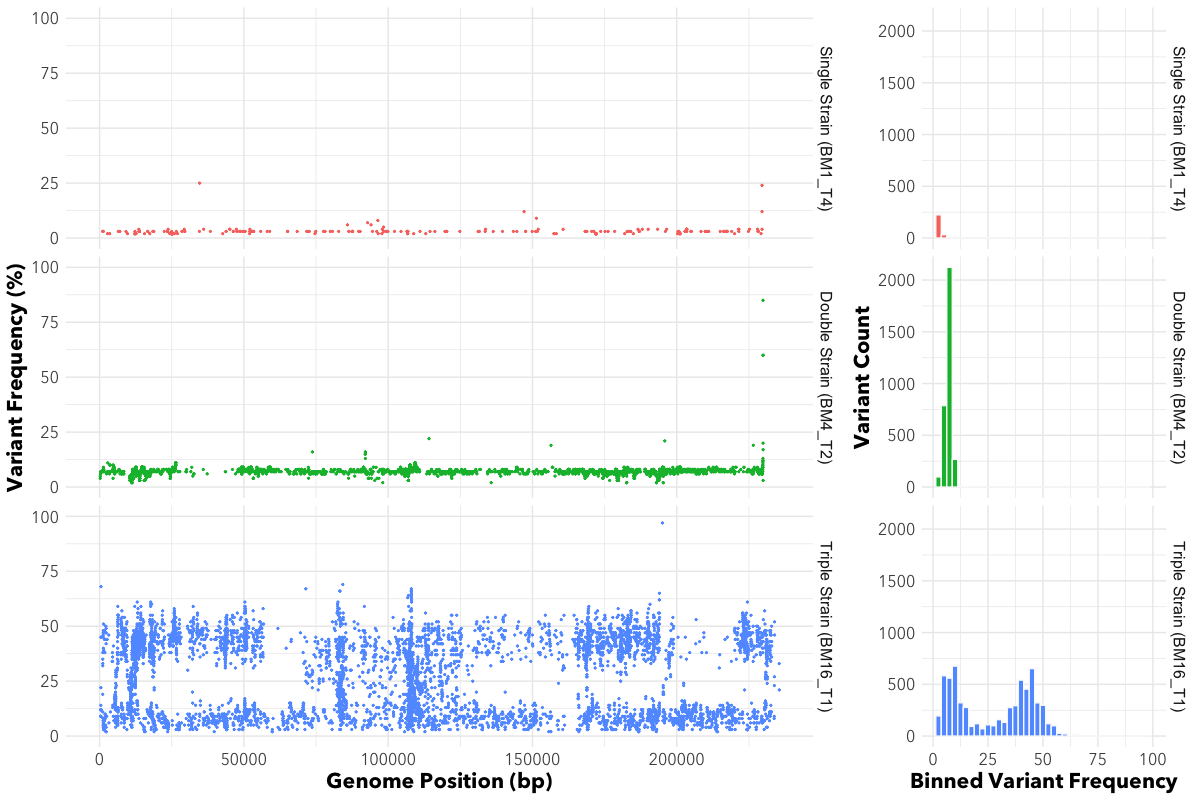


**Supplementary Figure 4.** Representative intrasample variant plot for datasets representing infection with 1, 2, or 3 strains. Each dot indicates a variant at a particular position in the genome supported by deduplicated reads. Each histogram bins the total number of SNPs present at a certain frequency.


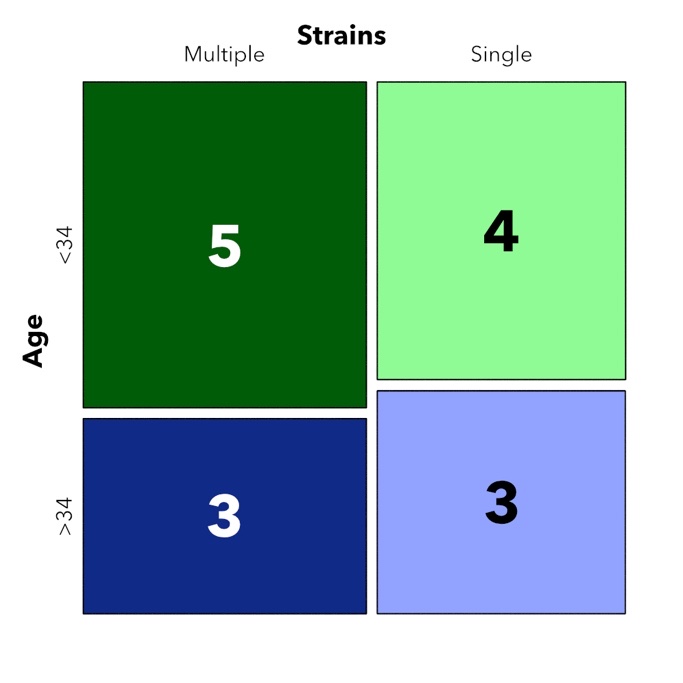

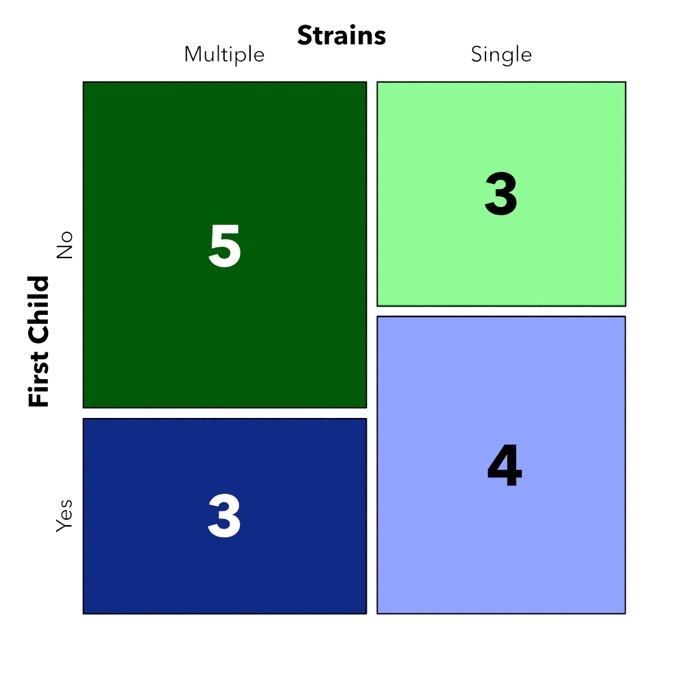


**Supplementary Figure 5.** Mosaic plot of contingency tables for number of strains (single or multiple) in relation to a) mother’s age (years) and b) whether the child was the first. No significant trend was found towards more multiple-strain infections for these variables.

**Supplementary Table 1.** (Separate Excel File) Full information on genotyping of all datasets with respective assembly statistics.
